# The Mast Cell–PAR2–TRP Axis: A Convergent Mechanism for Visceral Hypersensitivity Independent of Divergent Motility in IBS

**DOI:** 10.3390/biom16030469

**Published:** 2026-03-20

**Authors:** Kaiyue Deng, Jiazhen Cao, Zitong Wang, Jing He, Jialin Jia, Ru Nie, Xingbang Wang, Zhiqiang Dou, Zijian Liu, Yongzhi Deng, Tie Li

**Affiliations:** 1 Department of Acupuncture and Tuina, Changchun University of Chinese Medicine, Changchun 130117, China; 18943184582@126.com (K.D.); wzt100434131@126.com (Z.W.); hejing199801@163.com (J.H.); jiajialin0416@126.com (J.J.); nieru19981229@163.com (R.N.); wxbccucm@163.com (X.W.); douzhiqiang12345@163.com (Z.D.); 17604207415@163.com (Z.L.); 2 School of Nursing, Changchun University of Chinese Medicine, Changchun 130117, China; caojz@ccucm.edu.cn

**Keywords:** irritable bowel syndrome, visceral hypersensitivity, mast cell, PAR2, TRP

## Abstract

Most patients with irritable bowel syndrome have diarrhea or constipation, two opposite bowel habits. Although defecation habits represent opposing phenotypes, patients across all subtypes exhibit visceral hypersensitivity. This review explores the common pathway that causes visceral hypersensitivity: the mast cell–PAR2–TRP axis. The mechanism involves tryptase released by mast cells. Furthermore, tryptase activates PAR2, which sensitizes downstream TRP ion channels that conduct pain signals. The review also examines the factors leading to the formation of different fecal characteristics. In terms of treatment, this review also summarizes therapeutic agents targeting different components of this axis. Future pharmaceutical research should focus more on the mast cell–PAR2–TRP axis.

## 1. Introduction

Irritable bowel syndrome (IBS) is a common disorder of the gut–brain axis [1,2]. About 4.1% to 10.1% of the world’s population suffers from this disease. The affected population consists mainly of middle-aged and young adults. The prevalence of this disease is much higher in women than in men [3,4]. The disease imposes a significant socio-economic burden, with an impact on quality of life comparable to that of chronic organic diseases [5,6,7]. The Rome IV criteria indicate that recurrent abdominal pain changes with defecation as the core feature of IBS. In addition to the main symptom, the Bristol Stool Form Scale is used to evaluate stool consistency, according to which patients are divided into Constipation-predominant IBS (IBS-C), Diarrhea-predominant IBS (IBS-D) or mixed type (IBS-M) [8,9].

The bowel habits of IBS-C patients are diametrically opposed to those of IBS-D patients. IBS-C is characterized by delayed colonic transit. IBS-D is characterized by the acceleration of colonic transit [10,11]. Abdominal pain represents the cardinal symptom shared by patients across the spectrum of bowel habit abnormalities [12,13]. Although the colonic transit time can effectively predict stool form, it cannot measure the severity of abdominal pain [14]. Patients with different subtypes of IBS showed the same abdominal pain symptoms. Regardless of the subtype, the pain threshold of the rectum and colon is significantly reduced. This is mainly due to the visceral hypersensitivity (VH) [15]. Clinically, patients should be given antidiarrheal drugs or laxatives for different defecatory patterns. However, the application of drugs for the symptom of visceral hypersensitivity, which is a distressing symptom for patients, is not common.

VH is characterized by some allodynia, or exaggerated nociceptive response to painful stimuli [16,17]. VH is a core symptom of IBS, affecting 30–60% of patients [18,19]. Consequently, IBS research should expand its focus beyond the divergent mechanisms of altered defecation to prioritize the common signaling pathways underlying VH. Only through this integrated approach can we truly clarify the pain mechanism of IBS and lay the foundation for the development of general-purpose analgesic therapy. Various factors trigger the activation of mast cells (MCs), subsequently leading to their degranulation. The tryptase released upon degranulation activates protease-activated receptor 2 (PAR2) expressed on neuronal surfaces. After PAR2 activation, sensitization of TRP channels leads to a marked decrease in the response threshold of the peripheral nociceptors, thus inducing abdominal pain symptoms [20].

This review analyzes the literature on the mechanism of abdominal pain caused by VH in patients with IBS. The study shows that the mast cell–PAR2–TRP channel axis plays a pivotal role in the VH of IBS patients. Diverse stimuli transmit highly sensitive pain signals through this pathway. This review also distinguishes between pain pathways and motor pathways that affect intestinal peristalsis of different IBS subtypes. The respective representative mechanisms of different subtypes are clarified separately. Finally, we summarize the existing treatment targets in the signaling pathway and establish a mechanistic framework for the development of new therapies, which can effectively alleviate the visceral pain sensitivity of all IBS subtypes without compromising normal intestinal motor function.

## 2. The Convergent Pathway: The Mast Cell–PAR2–TRP Channels Axis in VH

### 2.1. Mast Cells and Tryptase: Initiators of This Axis

Mast cells are immune cells that regulate the immune function of intestinal mucosa [21,22]. Mast cell activation is one of the common pathological characteristics in the colon mucosa of all IBS subtype patients. Mast cell activation is a key initiator of VH, causing abdominal pain symptoms in IBS patients. A study using mast cell-deficient rats explored the role of these cells in TNBS-induced visceral hypersensitivity. Because this TNBS-based model is commonly used to mimic post-inflammatory visceral hypersensitivity and neuroimmune alterations relevant to IBS, particularly post-infectious IBS, it provides useful mechanistic insight, although it does not fully recapitulate the multifactorial clinical phenotype of human disease. The results showed that while TNBS induced significant hypersensitivity in control rats, it failed to elicit a similar response in rats lacking mast cells. This indicated that the pain threshold remained stable in the absence of mast cells, confirming their essential role in the development of VH [23]. However, at present, there is a contradiction in the statistical data of the article on the study of the number of mast cells in the colon mucosa of IBS patients [24]. The process of releasing inflammatory mediators by activated mast cells can more accurately reflect the pathological state of IBS than detecting changes in the total number of mast cells [25]. Mere quantification of the number of mast cells cannot predict the efficacy of IBS treatment. The activation state of mast cells is the key factor in determining the therapeutic effect and the progression of the disease [26,27].

Various stimuli can lead to the activation of mast cells in the intestine. Once mast cells are activated, they trigger an immune response. Simultaneously, it triggers pseudo-allergic reactions that closely mimic traditional immune responses. Food-specific IgG-associated responses have been proposed as a potential contributor to symptom generation in a subset of IBS patients, although their mechanistic role in mast cell activation remains incompletely established. This reaction is different from IgE-mediated rapid allergic reaction. Studies show that the level of IgG antibodies against specific foods in IBS patients is significantly increased. By avoiding such foods, patients experience a significant reduction in discomfort. This indicates that the complex formed by IgG antibodies and food ingredients can induce VH and pain. The mechanism may be that these complexes trigger the activation of immune cells such as mast cells, resulting in VH enhancement [28]. Furthermore, the underlying mechanisms of non-antibody-mediated pseudo-allergic reactions warrant further in-depth investigation. The study found that the number of MRGPRX2 receptors on the surface of colonic mast cells in IBS patients was higher than that in healthy people. This receptor recognizes non-immunologic stimuli such as spicy foods, certain medications, and the body’s own substance P. It can directly trigger rapid release of pain- and inflammation-inducing substances from mast cells without IgE antibody mediation. This mechanism partly explains why IBS patients exhibit heightened sensitivity to dietary and pharmaceutical stimuli [29]. Moreover, a research has proposed the local immune activation hypothesis. Aguilera-Lizarraga et al. demonstrated that intestinal infections can induce local IgE production against specific food antigens, triggering mast cell degranulation and sensitizing TRPV1 channels without inducing systemic allergic reactions [30]. Meanwhile, Fritscher-Ravens et al. used confocal laser endoscopy to observe in real time that IBS patients exhibit immediate widening of epithelial gaps and immune cell recruitment in the intestinal mucosa upon antigen exposure, supporting the concept that local mucosal antigen exposure can rapidly induce epithelial and immune responses in a subset of IBS patients [31]. Second is the regulation by host neuroendocrine factors. At the molecular level, psychological stress directly induces rapid degranulation and release of core mediators such as Tryptase by activating the CRF-R1 receptor signaling pathway on the surface of mast cells [32,33]. In addition to the aforementioned factors, estrogen is also one of the key factors inducing mast cell degranulation. Estrogen potentates the systemic stress response, thereby driving immune activation or compromising intestinal barrier integrity [34]. Estrogen works by activating the estrogen receptor (ER). ER activation exerts a dual effect, augmenting mast cell function while concurrently regulating the spatial orientation of degranulated mediators in the intestinal mucosa [35]. These estrogen-related effects may contribute to sex-related differences in visceral hypersensitivity and may partly help explain the higher prevalence of IBS in women.

Physical stimulation, metabolites and tissue damage signals damage the intestinal microenvironment. Mast cells play a key role in this process. From the perspective of physical signal regulation, the mechanically sensitive ion channel *Piezo1* induces intestinal mechanical traction stimulation. Subsequently, the cytokine IL-33 heightens the sensitivity of these channels to mechanical stimuli, thereby triggering mast cell degranulation and the release of mediators even during normal physiological traction [36]. From the perspective of metabolic signal regulation, excessive bile acids can promote mast cell-to-nociceptor signaling and induce NGF-related sensitization, thereby aggravating visceral pain [37]. Signal molecules released after tissue damage can also act on mast cells and participate in the regulation of pain pathways. HMGB1 can promote the production of a variety of inflammatory mediators and reactive oxygen in mast cells through the RAGE receptor. Evidence suggests that when the HMGB1 signaling pathway was inhibited, the activation effect of mast cells was also significantly weakened [38].

**Figure 1:**
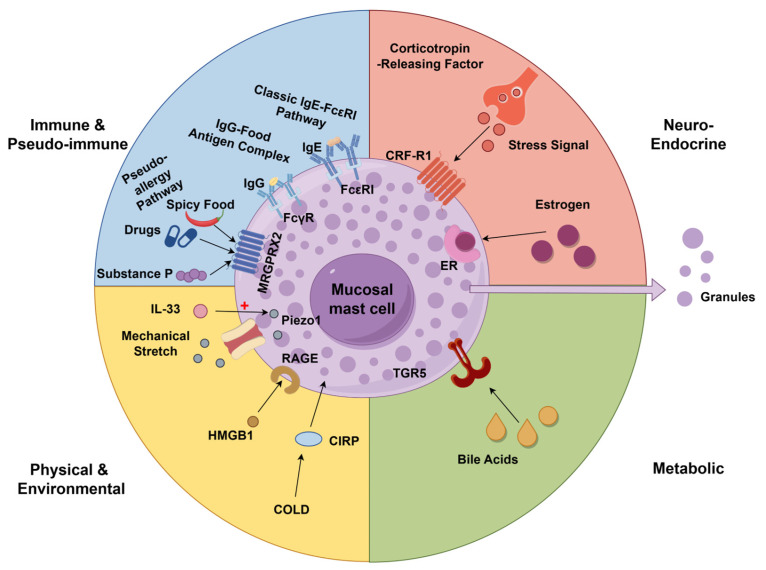
Multiple factors contributing to mast cell activation in IBS. This figure illustrates multiple stimuli that induce mast cell degranulation in the intestinal mucosa of IBS. Immune & pseudo-immune (upper left, blue): Canonical IgE-FcεRI pathway; IgG-food antigen complexes via FcγR; non-immunogenic stimuli (spicy food, drugs, substance P) via MRGPRX2. Neuroendocrine (upper right, red): Psychological stress signaling via CRF-R1; estrogen enhancing mast cell activity via ER. Physical & environmental (lower left, yellow): Mechanical stretch activating *Piezo1* (potentiated by IL-33); tissue damage signal HMGB1 via RAGE; cold stimulus CIRP via cold-sensitive pathways. Metabolic (lower right, green): Excess bile acids aggravating visceral pain through bile acid receptor-related mast cell–nerve signaling. These pathways collectively trigger mast cell granule release and contribute to visceral hypersensitivity. Arrows indicate activation or signaling pathways, and red plus signs (+) indicate promoting or enhancing effects. (Created in Figdraw. Kaiyue Deng. (2026) https://www.figdraw.com).

Cold-induced RNA binding protein (CIRP) is also closely related to mast cell activation and pain regulation. Experimental evidence demonstrates a significant upregulation of CIRP expression in murine models of IBS. The increase in CIRP activates mast cells, thus exacerbating intestinal barrier damage and development of VH. Suppression of CIRP expression not only inhibits mast cell activation but also restores intestinal function and attenuates VH [39]. The above-mentioned physiological, metabolic and damage-related signals lead to the activation of mast cells, and the mediators accompanying the degranulation process of mast cells work together to finally push the pain pathway to a more sensitive state. Various factors including immune triggers, neuroendocrine factors, and physical or metabolic stimuli that lead to the activation of mast cells are summarized in Figure 1.

### 2.2. PAR2: A Mediator of Signaling from Immune to Neural Signals

Among the mediators released by mast cells, tryptase is the main factor leading to the VH. It can activate mucosal immunity and transmit nerve signals [40]. It was observed that the tryptase released by mast cells in the mucosal biopsy supernatant of IBS patients can activate the intestinal nerve [41]. Follow-up experiments confirmed that the activation effect of colon biopsy supernatant on sensory neurons in IBS patients is positively related to their VH. The supernatant of VH patients showed a stronger neuronal activation effect, and the degree of activation was significantly related to the individual pain threshold [42]. And this process is usually related to the relationship between tryptase and PAR2. The process by which tryptase regulates neural excitability involves signaling through the specific receptor protease-activated receptor 2 (PAR2) to modulate gastrointestinal function [43].

PAR2 belongs to the G protein-coupled receptor family and is widely distributed on the surfaces of intestinal epithelial cells, immune cells, and primary afferent neurons [44,45]. Research shows that tryptase can induce hyperexcitability in submucosal neurons of guinea pigs by activating PAR2 [46]. Clinical studies have shown that IBS patients exhibit significantly elevated levels of mast cells and multiple neuropeptides, alongside markedly increased mRNA and protein levels of tryptase and PAR2 in colonic tissue [47]. This suggests that both may participate in the development of IBS symptoms by regulating neuropeptide release. Relevant studies indicate that PAR2 agonists are potent secretagogues for human colonic mast cells and may be implicated in the development of IBS in humans [48]. After the tryptase secreted by mast cells specifically cleaves the N-terminal extracellular domain of the PAR2 receptor, the previously concealed SLIGKV-NH_2_ sequence is exposed; this sequence then acts as an endogenous PAR2 agonist; upon binding to the receptor’s extracellular loop, it drives PAR2’s self-activation [49]. PAR2 can also promote the paracrine interaction between mast cells and eosinophils. Futagami et al. reported increased duodenal PAR2-positive inflammatory cell signals, particularly involving eosinophil-associated responses, in patients with functional dyspepsia–IBS overlap [50]. In a rat model used to reproduce stress-related and sensory abnormalities relevant to IBS, PAR2 gene deletion significantly improved VH, stress-related behavior, and colonic electrical activity [51]. This further confirms that PAR2 has a regulatory effect on VH, stress response and colon electrical activity in IBS animal models. The use of PAR2-specific antagonists or gene knockout technology to treat IBS rats can block the sensitization effect of colon biopsy fluid from IBS patients on dorsal root ganglia (DRG) neurons, confirming that PAR2 signals play a key role in regulating the excitability of IBS neurons [52,53].

Some IBS patients have symptoms of chronic abdominal pain. PAR2-mediated pain signals are biased and persistent, which leads to the possibility of chronic abdominal pain in IBS patients. This continuous transmission challenges the conventional paradigm limited to membrane signal conduction. Jimenez-Vargas et al. demonstrated that overactivated trypsin, elastase, and cathepsin S in the colonic mucosa of IBS patients can overactivated PAR2 receptors, thereby sensitizing colonic neurons to mechanical stimulation and contributing to persistent pain [54]. The study found that in transgenic colitis mice expressing fluorescently labeled PAR2, the receptor was internalized from the plasma membrane of colonic cells and redistributed to early endosomes, thereby promoting inflammation and enhancing pain sensitivity. However, in mice treated with PAR2 agonists, the intercellular permeability of colon epithelial cells increased, which promoted the release of pro-inflammatory cytokines in the colon segment [55]. The above molecular mechanism and fluorescence labeling data clarify the biological basis of IBS chronic abdominal pain and confirm that PAR2 is a promising target in analgesia and anti-inflammatory treatment.

### 2.3. Signal Coordination and Amplification Mechanisms in TRP Channels

Transient Receptor Potential (TRP) channels are a conserved superfamily of ligand-gated, non-selective cation channels, widely expressed in the gastrointestinal tract on primary afferent neurons, epithelial cells and immune cells [56]. These channels function as polymodal signal sensors, capable of converting thermal, mechanical, chemical and inflammatory stimuli into cellular cation influx and neuronal excitation, serving as a core hub for visceral pain signal transduction in the enteric nervous system [57]. Rather than directly initiating neuronal pain signals, PAR2 functions by modulating the activity of downstream ion channels to facilitate neuronal activation. TRP channels are the principal downstream ion channels through which PAR2 facilitates neuronal activation and visceral nociceptive signaling. The key factors affecting VH in IBS patients are three protein channels: TRPV1, TRPA1, and TRPV4 [58,59].

#### 2.3.1. TRPV1

TRPV1, primarily activated by capsaicin and noxious heat stimuli, is the most extensively studied subtype associated with IBS visceral hypersensitivity [60]. The expression level of TRPV1 protein in patients with IBS is significantly higher than that of healthy people, and this expression is positively related to the severity of abdominal pain symptoms [61]. This phenomenon is also found in patients with inflammatory bowel disease (IBD) with IBS symptoms. Studies have found that the number of TRPV1 immunoreactive fibers in the rectal sigmoid colon mucosa of IBS and IBD patients increased significantly, and it was directly proportional to the abdominal pain score [62]. This abnormal activation may be affected by epigenetic regulation. Studies have found that the level of microRNA-199 in the colon tissue of IBS patients is reduced, and this substance usually inhibits the production of TRPV1. The reduction in microRNA-199 leads to the excessive accumulation of TRPV1 in sensory neurons, thus causing pain [63]. However, although some IBS patients with VH did not show significant abnormalities in rectal TRPV1 mRNA or protein expression, they showed significantly enhanced pain perception of rectal capsaicin stimulation. This indicates that TRPV1 dysfunction may be caused by changes in receptor sensitivity rather than simple upregulation [64].

The sensitization of TRPV1 is the core mechanism of its transmission of pain signals. When PAR2 is activated by tryptase, it initiates the PKC and PKA kinase cascade reaction of G protein coupling, significantly enhancing the sensitivity of TRPV1 through phosphorylation modification [65,66]. After the TRPV1 receptor becomes abnormally sensitive, it produces a strong reaction even in the face of harmless stimuli such as normal intestinal peristalsis and metabolites. Subsequently, TRPV1 facilitates a massive influx of calcium ions into neurons, generating a surge in intracellular calcium signaling that ultimately exacerbates the hypersensitive pain response [67]. PAR2 also indirectly enhances the expression of TRPV1 by promoting the release of 5-HT. Research shows that 5-HT can activate the downstream signaling pathway through 5-HT2 and 5-HT4 receptors to enhance the ionic current induced by capsaicin and acidic stimulation, significantly improving the pain signaling ability of TRPV1 [68]. The visceral hypersensitivity caused by TRPV1 activation can also be mediated by the 5-HT pathway. Reducing the 5-HT level can reduce the excitability of TRPV1 in the dorsal root ganglion (DRG) neurons [69]. PAR2 releases a neurotransmitter called Calcitonin Gene-Related Peptide (CGRP) after activating intestinal neurons. CGRP inhibits the function of regulatory T cells, hinders the maintenance of immune homeostasis in the intestine, thus amplifying the inflammatory response, and triggers pain signals through PKA phosphorylation TRPV1, causing VH [70]. TRPV1 can also trigger the retrograde release of CGRP through axon reflex. It further attracts mast cells to gather and degranulate. The degranulation of mast cells continues to regulate the activity of TRPV1 through the action of PAR2, forming a vicious circle. The study found that TRPV1-specific antagonists can reverse this stress-induced pain response [71]. This further reveals the potential value of TRPV1 antagonists in the treatment of VH symptoms in IBS.

#### 2.3.2. TRPA1

Similar to TRPV1, TRPA1 is highly associated with IBS-related visceral pain, and the expression level of TRPA1 gene in the sigmoid colon of IBS patients was significantly higher than that in healthy people [72]. TRPV1 is more sensitive to thermal and acidic stimuli, while TRPA1 is more sensitive to mechanical pulling, cold stimulation and inflammatory substances. The focus of TRPA1 is to regulate the harmful substances produced by intestinal mechanical pulling, inflammatory reactions and cold stimulation, and converting these stimulation signals into pain and transmitting them to the brain [73,74,75]. The upstream PAR2 protein can enhance the sensitivity of TRPV1 and TRPA1 at the same time, thus expanding the range of perceived pain. This amplification effect amplifies noxious stimuli into intense pain signals, which is eventually transmitted to the brain to form chronic pain [76]. PAR2 can improve the reactivity of TRPA1 to mechanical stretching and oxidative stress. The mechanical pressure generated by fecal expansion in the intestine of IBS-C patients activate TRPA1. The study found that after the intestine is subjected to mechanical pressure, PAR2 degrades PIP2 on the cell membrane through the phospholipase C signaling pathway. This process weakens the inhibitory effect of TRPA1 and makes its channel easier to open, thus enhancing the pain signal [77]. Research demonstrated that the histamine activated and released by PAR2 enhances the sensitivity of TRPA1 through the H1 receptor, thus triggering a stronger calcium ion inflow reaction [78]. And the synergy of TRPA1 and TRPV1 can also continue to drive and amplify the pain signals transmitted to the central nervous system, eventually leading to chronic abdominal pain [79]. In rats subjected to chronic water avoidance stress, TRPV1 and TRPA1 were upregulated in colonic afferent dorsal root ganglia and were associated with stress-induced visceral hyperalgesia [80]. This stress-based model is mainly used to mimic gut–brain axis dysregulation and stress-induced visceral hypersensitivity relevant to IBS, rather than the full clinical syndrome of human IBS. In the context of cold stress, research demonstrates that the activation of specific signaling pathways promotes calcium ion influx, thereby heightening sensitivity to cold-induced abdominal pain. Blocking TRPA1 can eliminate this stress-induced sensitivity, indicating that TRPA1 and TRPV1 are the core targets of stress-induced intestinal pain sensitivity [75]. TRPA1 activation also releases CGRP together with TRPV1, causing intestinal vasodilation and promoting immune cell aggregation, thus further amplifying pain perception [81].

Unlike other proteins in the TRP channel family, although TRPA1 expression increases during colon inflammation, TRPA1 can also reduce the release of some pro-inflammatory substances and has a slight protective effect [82]. However, whether these subtle protective effects can be clinically translated into effective IBS therapies warrants further in-depth investigation. In addition, TRPA1 activation can promote the release of serotonin (5-HT) by intestinal epithelial cells, which not only enhances the sensitivity of TRPV1 but also regulates intestinal peristalsis [83]. This provides a new way to relieve constipation-related pain in IBS-C patients.

#### 2.3.3. TRPV4

But unlike TRPV1 and TRPA1, it is more sensitive to changes in intestinal osmotic pressure and temperature [84,85]. TRPV4 is distributed in intestinal sensory nerves, intestinal ganglia and vascular endothelial cells, which is involved in causing abdominal pain and intestinal movement abnormalities in IBS patients [86]. After TRPV4 is sensitized by PAR2 regulation, it mediates pain hypersensitivity and movement abnormalities [84]. Additionally, phosphorylation mediated by protein kinases PKC and PKA can potentiate TRPV4 activity, a mechanism analogous to the enhancement of nociceptive signaling through TRPV1 [87]. The sensitization effect of histamine and 5-HT on TRPV4, like the sensitization mechanism of TRPV1 and TRPA1 channels, further enhances the synergistic effect of TRP channels [88,89]. The difference is that TRPV4 can directly regulate intestinal motor function. When TRPV4 is activated, it promotes the release of nitric oxide (NO). Moreover, NO can inhibit colon muscle contraction and slow down intestinal peristalsis, thereby alleviating intestinal dysfunction related to IBS-D [90]. TRPV4 can also activate glial cells in the intestine, which further inhibit excessive intestinal contraction through calcium ion signal conduction and help restore normal intestinal rhythm [91]. PAR4 protein can reversibly inhibit TRPV4 activity, counteract the sensitization effect induced by PAR2, and maintain pain homeostasis. This mechanism also provides new ideas for the development of therapeutic drugs for IBS [92].

**Figure 2:**
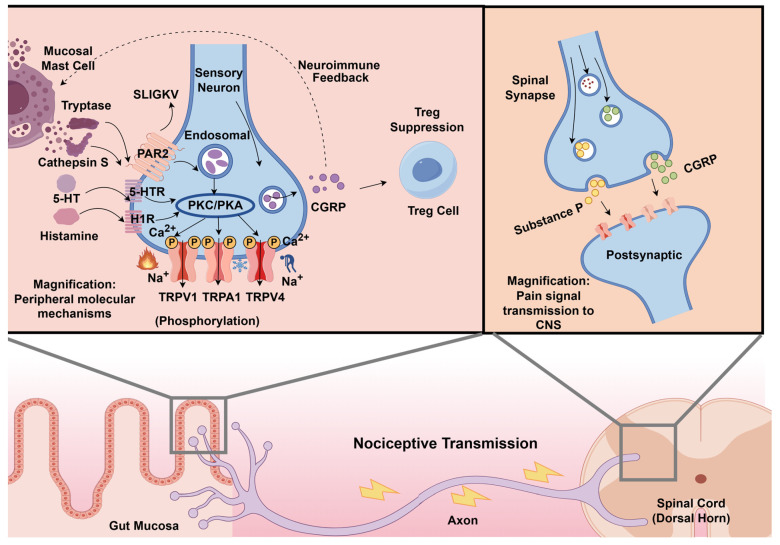
Mast Cell–PAR2–TRP Pathway and Its Signaling Mechanisms in Visceral Hypersensitivity of IBS. This diagram illustrates the integrated pathway from mucosal activation to central pain perception. Magnification: Peripheral Molecular Mechanisms (Left): Activated MCs release Tryptase, Cathepsin S, 5-HT, and Histamine. Tryptase cleaves PAR2, exposing the SLIGKV tethered ligand, which induces receptor self-activation and Endosomal internalization. PAR2 activates PKC/PKA kinase cascades, leading to the phosphorylation and sensitization of TRPV1, TRPA1, and TRPV4 channels. This results in enhanced Na^+^ and Ca^2+^ influx, causing neuronal hyperexcitability. Sensitized neurons release CGRP, which provides neuroimmune feedback and causes Treg Suppression, further amplifying the inflammatory response. Magnification: Pain Signal Transmission to CNS (Right): Nociceptive signals travel along the Axon from the Gut Mucosa to the Spinal Cord (Dorsal Horn), where the release of Substance P and CGRP at the Spinal Synapse transmits pain information to the Postsynaptic neuron for central processing. (Created in Figdraw. Kaiyue Deng. (2026) https://www.figdraw.com).

TRPV1, TRPA1 and TRPV4 share a common regulatory pathway in response to different types of stimuli, which together constitute the core regulatory network of visceral pain and intestinal dysfunction in IBS [93]. Clinical and ex vivo human data provide important, although still incomplete, support for the mast cell–PAR2–TRP axis in IBS. Mucosal biopsy supernatants from IBS patients have been shown to activate intestinal and sensory neurons, and the magnitude of this neuronal activation is associated with visceral hypersensitivity, supporting a role for altered mucosa–nerve signaling in human disease [41,42]. In addition, clinical studies have shown elevated tryptase and PAR2 mRNA and protein expression in colonic tissues from IBS patients [47]. Human tissue studies also demonstrate increased TRPV1 expression in IBS and a positive correlation with abdominal pain severity [61]. However, direct demonstration of the complete tryptase–PAR2–TRP signaling sequence within human IBS tissue remains limited, and much of the mechanistic continuity is still inferred from ex vivo neuronal assays and animal models. Therefore, the mast cell–PAR2–TRP axis is best viewed as a convergent and biologically plausible framework for visceral hypersensitivity in IBS, rather than a fully established and subtype-exclusive mechanism. The integrated signaling of the mast cell–PAR2–TRP axis and the subsequent pain transmission to the central nervous system are illustrated in Figure 2.

## 3. Mechanisms Underlying Divergent Defecation Phenotypes in IBS-D and IBS-C

**Figure 3:**
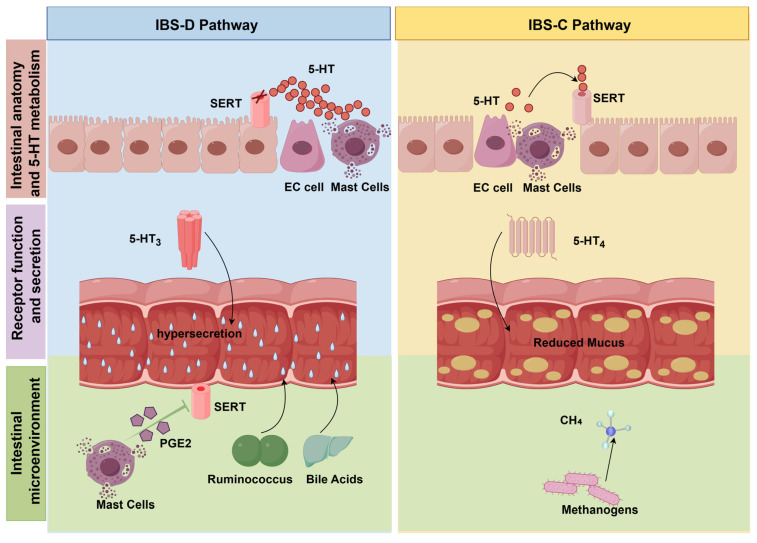
Divergent driving factors of stool form and motility in IBS-D and IBS-C. This figure illustrates the distinct pathophysiological mechanisms leading to diarrhea-predominant (IBS-D) and constipation-predominant (IBS-C) phenotypes. Intestinal Anatomy and 5-HT Metabolism (Top): In the IBS-D pathway (left), SERT function is impaired, leading to excessive 5-HT accumulation secreted by EC cells and mast cells. In contrast, the IBS-C pathway (right) is characterized by reduced 5-HT availability or overactive SERT reabsorption. Receptor Function and Secretion (Middle): Excess 5-HT in IBS-D activates 5-HT_3_ receptors, triggering hypersecretion and rapid transit. In IBS-C, weakened 5-HT_4_ receptor signaling results in reduced mucus secretion and lubrication, delaying transit. Intestinal Microenvironment (Bottom): In IBS-D, mast cell-derived PGE2 inhibits SERT activity, whereas PGD2, the predominant prostanoid generated during mast cell activation, may further promote epithelial secretion and smooth muscle contraction. Together with Ruminococcus and excessive bile acids, these factors accelerate intestinal transit. In IBS-C, the overgrowth of methanogens produces CH_4_, which inhibits smooth muscle contraction and exacerbates constipation. (Created in Figdraw. Kaiyue Deng. (2026) https://www.figdraw.com).

The mast cell–PAR2–TRP axis, described in the preceding sections, constitutes the core shared mechanism mediating VH, a common pathological feature in both IBS-D and IBS-C. However, this shared pain pathway does not explain the diametrically opposed defecation phenotypes of the two subtypes. While the mechanisms underlying VH are largely conserved across IBS subtypes, the pathways regulating intestinal motility and epithelial secretion differ substantially between IBS-D and IBS-C. These subtype-specific differences are driven mainly by alterations in the 5-HT system, gut microbiota, and other modulators of intestinal motility and secretion, which together shape the divergent clinical manifestations of diarrhea and constipation (Figure 3). In this framework, the mast cell–PAR2–TRP axis is best understood as a shared nociceptive platform underlying VH, whereas subtype-specific stool patterns are more directly shaped by differential regulation of intestinal motility, epithelial secretion, and luminal hydration by factors such as 5-HT signaling, gut microbiota, bile acid metabolism, and related mediators.

### 3.1. Major Drivers of the Diarrheal Phenotype in IBS-D

Beyond the shared VH pathway, IBS-D is characterized predominantly by mechanisms that accelerate intestinal transit and enhance epithelial secretion, thereby producing diarrhea. In addition to abdominal pain, the symptoms of IBS-D are also caused by rapid intestinal peristalsis leading to diarrhea. This is mainly caused by 5-HT metabolic disorders, gut microbiota imbalance and bile acid metabolism abnormalities.

Mast cells and Enterochromaffin cells (EC cells) in the intestine produce 5-HT. 5-HT is taken up by 5-HT transporter (SERT) after secretion. SERT recycles excess 5-HT to achieve a balance of 5-HT content in the body. If the equilibrium state of 5-HT is disturbed, it may cause disorders of intestinal motor function [94]. The SERT function in the intestine of IBS-D patients is impaired. This damage may be manifested by a decrease in SERT content or a decrease in its transport efficiency. Damage to SERT in IBS-D patients leads to more 5-HT content in the intestine, thus stimulating intestinal nerves [95]. Such stimulation accelerates the contractile frequency of intestinal smooth muscles, resulting in truncated transit time and inadequate water absorption, which ultimately culminates in watery stools [96,97]. Kumar et al. compared the serotonin transporter gene SLC6A4 between IBS patients and healthy controls and found that its polymorphism was associated with abnormal SERT function in IBS-D [98]. Based on this mechanism, some studies have improved intestinal peristalsis disorders and alleviated the diarrhea symptoms of IBS-D patients by regulating SERT and 5-HT signaling pathways to enhance the transport function of SERT. Further research found that the 5-HT_3_ receptor subtype of the 5-HT receptor family are key targets for IBS-D pain and diarrhea symptoms [99]. The clinical guidelines also point out that the use of specific drugs to inhibit excessive 5-HT3 can improve IBS-D diarrhea symptoms [100]. The increase in the level of specific gut microbiota may also cause abnormal recovery of 5-HT_3_ receptors and cause diarrhea. Ruminococcus can aggravate diarrhea symptoms by promoting 5-HT biosynthesis [101,102]. Its metabolites interact with bile acid to further accelerate intestinal peristalsis. Gao et al. showed that the gut microbiota can activate colonic mast cells, promoting the release of PGE2. PGE2 is a key inflammatory mediator that inhibits SERT activity, leading to 5-HT accumulation and aggravating diarrhea in IBS-D patients [103]. At the same time, activated mast cells also release PGD2, which is classically regarded as the predominant prostanoid generated during mast cell activation [104]. PGD2, together with PGE2, may also contribute to diarrheal symptoms. Specifically, PGD2 promotes chloride and water secretion via the DP1 receptor [105] and may further enhance motility by activating enteric cholinergic motor neurons [104]. Together, these distinct mechanisms accelerate intestinal transit and increase fecal water content.

In addition, abnormal bile acid metabolism may also induce diarrhea symptoms in patients with IBS-D. Clinical data show that 25% to 50% of IBS-D patients have intestinal bile acid absorption disorders. Excessive bile acid changes the osmotic pressure in the intestinal cavity after staying in the colon. Under the action of osmotic pressure, the moisture in the blood vessels of the intestinal wall is introduced into the intestinal cavity and intestinal peristalsis is accelerated, eventually causing diarrhea [106].

### 3.2. Major Drivers of the Constipated Phenotype in IBS-C

In contrast to IBS-D, IBS-C is characterized predominantly by mechanisms that reduce intestinal propulsion and impair luminal lubrication, thereby leading to constipation. The symptoms of IBS-C patients are constipation caused by slow intestinal peristalsis. The main cause is also related to 5-HT. The decrease in the content of 5-HT in the intestine causes the intestinal nerves to be unable to obtain sufficient stimulation signals. Or too high SERT activity leads to the rapid reabsorption of 5-HT, so that the intestinal nerves cannot be fully stimulated. Without sufficient stimulation, the intestine is unable to contract normally, and the intestinal transmission function slows down, which delays the discharge of contents, eventually causing constipation symptoms [107,108].

The functional differences of different subtypes in the 5-HT receptor family are the key features that distinguish the pathogenesis of IBS-D and IBS-C. The pathophysiological process of IBS-C mainly depends on the functional regulation of 5-HT_4_ receptors. When the signal of 5-HT_4_ receptor is weakened, it leads to insufficient mucus secreted by the intestinal mucosa. If the intestinal cavity environment is dry, the lubrication of the intestinal contents is reduced. Feces become dry due to lack of water and difficult to pass through the intestines, eventually causing constipation symptoms [109]. Clinical guidelines suggest that IBS-C patients can use 5-HT_4_ agonists to restore bowel movement rhythm. This kind of drug activates 5-HT_4_ receptors, thus regulating intestinal motility to relieve constipation symptoms [110,111].

The inhibitory effect of gut microbiota can also cause constipation symptoms in IBS-C patients. The methane produced by the proliferation of methane-producing bacteria in the patient’s intestine inhibits the peristalsis of the intestinal smooth muscle [112]. Altered 5-HT signaling and methane-producing microbiota may together contribute to delayed intestinal transit in IBS-C [113,114]. Under this mechanism, increasing the number of beneficial bacteria can promote the synthesis of 5-HT and improve constipation symptoms. Specifically, Lactobacillus plantarum AR495 participates in tryptophan metabolism, thereby promoting 5-HT synthesis within intestinal EC cells [115]. This is the clinical idea of improving IBS-C patients through 5-HT.

Taken together, these findings indicate that IBS-D and IBS-C share a common mechanism of visceral pain, but diverge in the regulatory pathways controlling intestinal motility and epithelial secretion, which ultimately determine their opposite defecation phenotypes.

## 4. Therapeutic Implications: Targeting the Axis

The mast cell–PAR2–TRP axis represents a paradigm shift in the treatment of VH. Targeted therapeutic agents can be developed against different sites along this axis. These therapies are designed to uncouple abdominal pain from intestinal motility, focusing instead on analgesic approaches effective across all IBS subtypes. We can categorize current and emerging interventions based on their roles in the signaling cascade (Table 1).

### 4.1. Upstream Intervention: Mast Cell-Targeted Therapy

In the mast cell–PAR2–TRP channel signaling pathway, mast cells are in the upstream position. The activation and degranulation of these cells trigger the transduction of downstream signaling pathways, ultimately leading to the development of VH in IBS patients. Therefore, stabilizing mast cells through pharmacological means to reduce activation and degranulation can improve the VH symptoms of IBS patients.

Ketotifen is a commonly used mast cell stabilizer at present. It has been confirmed that ketotifen can enhance the body’s tolerance to visceral pain, thus relieving abdominal discomfort [116]. Ketotifen relieves pain by reducing nerve sensitivity without affecting intestinal peristalsis. A randomized trial of IBS-D patients confirmed that ketotifen can reduce the count of mast cells at the end of the ileum to relieve abdominal pain symptoms [117]. Ketotifen also modulates the activity of extra-rectal mast cells and attenuates their degranulation, thereby alleviating VH symptoms in IBS-D patients [118]. The scope of application of another mast cell stabilizer, Disodium Cromoglycate (DSCG), in IBS has also been confirmed. DSCG can have a protective effect on the intestinal mucosa of IBS-D patients. Research confirms that oral DSCG can improve the intestinal pain of IBS patients. The mechanism is to inhibit the activity of cells in the ileum, thus reducing the release of tryptase [119]. This upstream intervention provides a new treatment option for patients with irritable bowel syndrome who do not respond to conventional antispasmodics.

In addition to these mast cell stabilizers, there are also some drugs that indirectly block the release of mast cell degranulation and release mediators [120]. The peripheral H1 receptor antagonist Ebastine can effectively relieve visceral pain in IBS patients and has good safety characteristics. Randomized controlled trials show that Ebastine antagonizes H1 receptors to intercept histamine-mediated signaling, thereby attenuating the nociceptive drive from mast cells and preventing downstream activation of PAR2 and TRP channels [121]. The peripheral restrictive cannabinoid receptor 2 (CB2) agonist Olorinab can also indirectly act on mast cells. Olorinab can bind to CB2 receptors on mast cells to inhibit the activation and degranulation of intestinal mast cells. However, translating these peripheral targets into clinical success remains challenging. For example, while the peripheral CB2 agonist Olorinab showed initial promise, it recently failed to meet its primary endpoint for abdominal pain reduction in a Phase 2b clinical trial (CAPTIVATE), highlighting the complexity of neuro-immune modulation in human IBS [122]. α2δ ligand Pregabalin is also a common drug for the treatment of IBS visceral pain [123]. Clinical trials have confirmed that Pregabalin can significantly relieve abdominal pain symptoms in patients with IBS by indirectly regulating mast cell-related pain pathways [124]. Pregabalin can regulate the voltage-gated calcium channel activity on central and peripheral neurons. This regulation can inhibit the release of pain transmitters such as Substance P and CGRP from intestinal sensory nerve endings. After the reduction in pain transmitters, it can indirectly inhibit the continuous activation and degranulation of mast cells, and finally improve the phenomenon of reducing the colon pain threshold [125,126]. Despite these positive findings, the broad clinical application of traditional mast cell stabilizers in IBS is still limited by the lack of large-scale, multi-center Phase 3 clinical trials.

### 4.2. Midstream Intervention: PAR2 Targeted Therapy

As an intermediate component of the mast cell–PAR2–TRP axis, PAR2 receives protease signals released by mast cells and transmits them to downstream TRP channels, ultimately triggering visceral hypersensitivity. PAR2 targeted therapy has become a key intervention point for connecting upstream mast cells and downstream TRP channels. PAR2 targeted therapy mainly activates PAR2 by blocking protease or directly inhibits the role of PAR2 receptor.

Excessive protease causes PAR2 to destroy the colon epithelial barrier and stimulate downstream TRP channels to cause pain. Tryptase is a key protease involved in the degranulation of mast cells and can activate PAR2 receptors. Tryptase inhibitors can block the activation of PAR2 from the source. Studies have confirmed that the serine protease activity in the fecal supernatant of IBS patients is about three times that of healthy people [127,128]. Intraperitoneal injection of Nafamostat or the new inhibitor UAMC-00050 blocks the activation of PAR2 and its downstream signaling pathway by inhibiting serine protease activity [129,130]. Local colonic administration of the serine protease inhibitor UAMC-00050 normalized visceral hypersensitivity in a post-inflammatory rat model [131]. These targeted tryptase inhibitors inhibit the transmission of pain signals to downstream TRP channels by inhibiting PAR2, thus obtaining a more comprehensive therapeutic effect on VH in IBS patients [132,133].

In addition to protease inhibitors, there are also antagonists that can directly act on PAR2 receptors. Research shows that the ligand binding site of PAR2 is shielded by the outer ring structure of the cell, and it is difficult for traditional small molecule drugs to contact this site [134]. Furthermore, many currently available specific PAR2 antagonists are peptide-based. These agents typically suffer from poor oral bioavailability and rapid enzymatic degradation in the gastrointestinal tract. This significantly limits their clinical feasibility as oral therapeutics [135]. Nevertheless, basic research has developed PAR2 antagonists. It has been observed that PAR2 antagonism can significantly reduce visceral hypersensitivity. In a PI-IBS mouse model, PAR2 antagonist treatment also normalized increased intestinal permeability and improved tight-junction-related barrier abnormalities [136]. However, some studies believe that PAR2 activation can promote intestinal secretion and peristalsis. Therefore, the use of PAR2 antagonists alone may aggravate the constipation symptoms of IBS-C patients. However, PAR2 antagonists remain investigational in IBS and currently lack guideline endorsement for routine clinical use. Therefore, their therapeutic value should be interpreted as preclinical or translational rather than established clinical practice.

### 4.3. Downstream Intervention: TRP Channel-Targeted Therapy

TRP channels are located downstream of the mast cell–PAR2–TRP axis and are responsible for subsequent pain signal transmission. Therefore, drugs targeting TRP channels can directly block abnormal signaling at the end of this pathway, thereby alleviating VH symptoms in IBS patients. TRP channel-targeted drugs mostly focus on subtypes related to intestinal nerves, such as TRPV1 and TRPA1. These targets can avoid the side effects of the central nervous system while regulating the function of the pathway.

Although TRP channels are attractive targets for visceral analgesia in IBS, some members of this family, particularly TRPV1, also participate in thermoregulation. Consequently, systemic TRP antagonists can cause severe side effects such as high fever [137]. Because of this, early clinical trials of these drugs failed. Currently, drug development no longer focuses on systemic antagonism. Instead, new therapies aim to use localized delivery or indirect modulation. Fast-acting TRPV1 channel agonists such as Palvanil can induce structural changes to TRPV1 receptors, thus preventing them from receiving and transmitting abnormal signals [138]. The strategy of this kind of drug is that after the TRPV1 channel is continuously activated with low intensity, it activates its own desensitization mechanism. The desensitized TRPV1 channel can no longer be opened for hours or even days even if it is stimulated, resulting in pain. This method can avoid the side effects of high fever of antagonists. Furthermore, this desensitization is reversible and does not compromise the structural integrity or permanently impair the target channel. Once the intestinal microenvironment normalizes, TRPV1 channel function is gradually restored, thereby preserving its essential physiological roles.

Beyond agonist-induced desensitization, TRP channel activity can be modulated indirectly through alternative signaling pathways to circumvent systemic side effects. The NHE3 inhibitor Tenapanor can treat IBS-C patients through multiple pathways to regulate TRPV1 channel function [139]. The strategy of this drug is to restore the abnormally elevated TRPV1 signal to normal by reducing inflammatory factors and antigen stimulation. The guanylate cyclase-C (GC-C) agonist linaclotide also indirectly modulates TRP channel-related nociceptive signaling. After Linaclotide activates GC-C, intestinal epithelial cells synthesize and release cyclic guanosine monophosphate (cGMP), resulting in an increase in the level of cGMP inside and outside the cell. Extracellular cGMP causes some TRP channels to sink from the cell membrane, reducing the number of channels that can be activated on the membrane, while reducing the response threshold of the remaining channels to stimulation, so that they no longer overreact to mild inflammatory stimulation in the intestine [140]. Clinical studies have also confirmed that Linaclotide can improve the severity of abdominal pain and constipation symptoms in IBS-C patients by regulating TRP channel-related signaling [141,142,143]. Although linaclotide has demonstrated clinical benefit in IBS-C, off-label use and non-standard prescribing patterns have been reported in several European countries. Such non-standard drugs may interfere with the normal physiological functions of intestinal TRP channels. Accordingly, in clinical practice, linaclotide should be used according to approved IBS-C indications, prescribed dosages, and recommended treatment durations [144].

Certain natural products can also target visceral hypersensitivity in IBS through TRP channels. The advantage of natural products lies in their ability to mildly regulate TRP channels with relatively high safety. The traditional herbal mixture Banhasasim-tang can regulate the TRP channel subtype TRPA1 as well as NaV1.5 and NaV1.7 channels, suppressing abnormal pain signals and relieving symptoms in IBS patients [145]. Atractylodes macrocephala Koidz. alleviates IBS-like symptoms by modulating TRPV1 channel activity in a zymosan-induced mouse model of IBS [146]. This model reproduces selected features such as diarrhea-predominant bowel dysfunction and visceral hypersensitivity, but does not fully reflect the complexity of human IBS. Ginger reduces intestinal hypersensitivity in IBS patients by inhibiting pro-inflammatory responses and decreasing the activation of TRPV1 and TRPA1 channels by inflammatory mediators [147].

In conclusion, the mast cell–PAR2–TRP axis provides a biologically plausible mechanistic framework and a potential therapeutic entry point for visceral hypersensitivity. However, translating this mechanism into clinical drugs still faces pharmacokinetic and safety challenges. Future therapies must avoid systemic exposure to improve clinical feasibility. Drug development should focus on gut-restricted delivery, stable non-peptide structures, and indirect modulation. In addition, clinical trials should use mucosal immune biomarkers to classify patients. This approach may complement traditional stool-based subtyping. It helps identify the specific IBS patients who will benefit most from these targeted therapies.

## 5. Conclusions

This paper proposes that despite the existence of different subtypes of IBS they may converge on a shared pain pathway, leading to visceral hypersensitivity. This pathway is the mast cell–PAR2–TRP axis. This study enumerates the causes of mast cell degranulation. Mast cell degranulation releases tryptase, activating PAR2 receptors on neurons. PAR2 activation sensitizes downstream TRP ion channels. Once sensitized, even normal intestinal motility may be perceived by the brain as pain. Based on this framework, the study summarizes representative therapeutic agents targeting each component of the mast cell–PAR2–TRP pathway. Future drug development may focus on this axis to inform broadly applicable analgesic strategies for IBS. Such medications may help alleviate visceral pain while minimizing disruption of normal intestinal motility.

## Figures and Tables

**Table 1:** Summary of therapeutic agents targeting the mast cell–PAR2–TRP axis.

Level	Agent	Mechanism of Action	Therapeutic Effect & Clinical Utility
Upstream: Mast Cells	Ketotifen	Stabilizes mast cells and reduces neuronal sensitivity.	Increases visceral pain tolerance, though broad application lacks large-scale Phase 3 validation.
	Disodium Cromoglycate	Inhibits ileal cell activity and tryptase release.	Provides mucosal protection and alleviates intestinal pain in IBS-D.
	Ebastine	Antagonizes peripheral H1 receptors.	Intercepts histamine signaling to prevent downstream axis activation.
	Olorinab	Activates peripheral CB2 receptors on mast cells.	Inhibits mast cell degranulation preclinically, but failed to meet primary endpoint.
	Pregabalin	Regulates α2δ subunits of voltage-gated calcium channels.	Reduces Substance P and CGRP release, indirectly inhibiting mast cell activation.
Midstream: PAR2	Nafamostat/UAMC-00050	Inhibits serine protease activity.	Blocks the activation of the PAR2 signaling pathway from the source.
	UAMC-00050	Local colonic serine protease inhibition.	Normalizes post-inflammatory visceral hypersensitivity in rats.
	PAR2 antagonist	Peptide-based PAR2 inhibitory strategy.	Reduces hypersensitivity preclinically; however, its peptide structure limits oral bioavailability.
Downstream: TRP Channels	Palvanil	Induces structural changes in TRPV1 receptors.	Triggers reversible desensitization to block pain signals, aiming to avoid systemic side effects like hyperthermia.
	Tenapanor	Inhibits NHE3 to modulate TRPV1 function.	Reduces inflammatory factors and restores normal TRPV1 signaling.
	Linaclotide	Activates GC-C to increase intracellular and extracellular cGMP.	Induces TRP channel internalization, reducing the number of active channels on the membrane.
	Natural Products	Modulate TRP subtypes such as TRPV1 and TRPA1.	Provide mild regulation of ion channels with high safety for analgesia.

## Data Availability

No new data were created or analyzed in this study. Data sharing is not applicable.

## Abbreviations

Abbreviation	Full Term
5-HT	5-Hydroxytryptamine
CB2	Cannabinoid receptor 2
CGRP	Calcitonin Gene-Related Peptide
cGMP	Cyclic guanosine 3’,5’-monophosphate
CIRP	Cold-induced RNA binding protein
CRF-R1	Corticotropin-releasing factor receptor 1
DRG	Dorsal root ganglia
DSCG	Disodium Cromoglycate
EC cells	Enterochromaffin cells
ER	Estrogen receptor
GC-C	Guanylate cyclase-C
HMGB1	High mobility group box 1
IBD	Inflammatory bowel disease
IBS	Irritable bowel syndrome
IBS-C	Constipation-predominant IBS
IBS-D	Diarrhea-predominant IBS
IBS-M	Mixed type IBS
MCs	Mast cells
MRGPRX2	Mas-related G protein-coupled receptor member X2
NGF	Nerve growth factor
NO	Nitric oxide
PAR2	Protease-activated receptor 2
PGE2	Prostaglandin E2
PIP2	Phosphatidylinositol 4,5-bisphosphate
RAGE	Receptor for advanced glycation end products
SERT	5-HT transporter
TNBS	2,4,6-trinitrobenzene sulfonic acid
TRP	Transient Receptor Potential
TRPA1	Transient receptor potential ankyrin 1
TRPV1	Transient receptor potential vanilloid 1
TRPV4	Transient receptor potential vanilloid 4
VH	Visceral hypersensitivity
